# Timing Anti-PD-L1 Checkpoint Blockade Immunotherapy to Enhance Tumor Irradiation

**DOI:** 10.3390/cancers17030391

**Published:** 2025-01-24

**Authors:** Steve Seung-Young Lee, Joanna Pagacz, Sera Averbek, David Scholten, Yue Liu, Stephen J. Kron

**Affiliations:** 1 Department of Pharmaceutical Sciences, College of Pharmacy, University of Illinois Chicago, Chicago, IL 60612, USA; 2 Department of Molecular Genetics and Cell Biology, University of Chicago, Chicago, IL 60637, USA; pagaczj@uchicago.edu (J.P.); saverbek@uchicago.edu (S.A.); dgscpb@yahoo.com (D.S.); yuel.chicago@gmail.com (Y.L.)

**Keywords:** immune checkpoint inhibitor, radiotherapy/radioimmunotherapy, combination immunotherapy

## Abstract

**Background:** The ability of radiotherapy (RT) to drive anti-tumor immunity is limited by adaptive resistance. While RT induces inflammation and recruits activated tumor-infiltrating lymphocytes (TILs), including cytotoxic T lymphocytes (CTLs), the resulting radiation- and IFNγ-dependent PD-L1 expression restores an immunosuppressed tumor microenvironment. Unleashing an effective anti-tumor response may require the precise sequencing of RT and checkpoint blockade immunotherapy (CBI) to block PD-L1 signaling before it can mediate its suppressive effects. **Methods:** Flank tumors formed in BALB/c mice with syngeneic CT26 colon or 4T1 mammary carcinoma cells were treated with otherwise ineffective doses of ionizing radiation (10 Gy) followed by CBI (0.2 mg anti-PD-L1, i.v.) after 0, 1, 3, 5, or 7 days, comparing tumor response. Anti-PD-L1 delivery was measured by fluorescence, TILs by flow cytometry and immunofluorescence, PD-L1 expression by immunohistochemistry, and tumor size by calipers. **Results:** In both CT26 and 4T1 tumors, 10 Gy alone resulted in a transient growth delay associated with infiltrating CTLs peaking at 3 days and PD-L1 at 5 days. CTLs returned to baseline after 7 days, consistent with adaptive resistance. Anti-PD-L1 failed to potentiate radiation except when injected 5 days after 10 Gy, which prevented CTL depletion and led to tumor elimination. Potentially contributing to compound effects, anti-PD-L1 penetrated tumors and bound PD-L1 more efficiently after irradiation. **Conclusions:** Optimal timing to exploit radiation-induced permeability to enhance CBI delivery and interrupt adaptive resistance by blocking PD-L1 as it peaks may offer a general strategy to enhance external beam radiotherapy by protecting activated TILs and potentiating anti-tumor immune response.

## Simple Summary

In this study, we performed anti-PD-L1 antibody therapy in preclinical mouse tumor models at different time points following single-dose ionizing radiation exposure to local tumors. We observed significant suppression of tumor growth in the group treated with anti-PD-L1 antibody at 5 days post-tumor irradiation. Longitudinal molecular and imaging assays of irradiated tumor tissues showed the largest number of tumor-infiltrating cytotoxic T lymphocytes (CTLs) and the highest expression of PD-L1 at 3 and 5 days post-10 Gy radiation, respectively. Systemic anti-PD-L1 therapy at 5 days post-radiation offered enhanced delivery of the therapeutic antibody to the tumor through damaged, leaky blood vessels and upregulated PD-L1 expression on cancer cells, resulting in the induction of strong anti-cancer immune response. The in vivo and in vitro works suggest that the IFNgamma from the CTL infiltrates is responsible for most of the PD-L1 expression at 5 days, leading to rebound immunosuppression. The anti-PD-L1 antibody therapy is thus able to interrupt the negative feedback and allow the CTLs to persist and eliminate the tumors.

## 1. Introduction

The binding of programmed cell death protein 1 receptor PD-1 (CD279), expressed by cytotoxic T lymphocytes (CTLs) and other immune effectors, to its ligand PD-L1 (B7-H1, CD274), expressed by myeloid and cancer cells in the tumor microenvironment (TME), limits CTL expansion, activation, cytotoxicity, and survival, driving immune evasion [1,2]. This interaction constitutes a major mechanism of immune checkpoint signaling exploited by tumors to suppress anti-tumor immunity. Multiple checkpoint blockade immunotherapy (CBI) antibodies are now approved that disrupt PD-1/PD-L1 signaling to restore CTL function in PD-L1-expressing tumors, achieving significant clinical responses in a subset of patients [3,4]. However, the response rate remains limited, particularly in tumors with low PD-L1 expression or immunologically “cold” phenotypes, underscoring the need for combinatory approaches to enhance efficacy. A complementary use for CBI may be in combination with external beam radiotherapy (RT) [5,6] and other treatments [7,8] that induce PD-L1 in efforts to overcome adaptive resistance.

Radiotherapy not only directly induces DNA damage leading to tumor cell death but also serves as a potent immunomodulatory tool by promoting immunogenic cell death (ICD) and releasing proinflammatory signals [9,10]. These signals recruit and activate antigen-presenting cells (APCs) and stimulate effector T cell responses, providing RT with vaccine-like effects. Despite these potential benefits, the immunogenic effects of RT are typically self-limiting. Activated T cells release IFNγ in the tumor, upregulating PD-L1 expression on tumor and immune cells, thereby driving rebound immunosuppression and adaptive resistance [11,12]. This dynamic interplay between RT and immune checkpoint signaling prompted investigations into the synergistic potential of combining RT with PD-1/PD-L1 blockade. Preclinical studies demonstrated that such combinations can potentiate anti-tumor immune responses; however, translation to the clinic has been met with challenges, such as identifying optimal dosing schedules, treatment sequences, and patient subsets likely to benefit [13,14].

In addition to its cytotoxic and immune-modulating effects, RT transiently disrupts the tumor microvascular permeability barrier [15,16], facilitating the extravasation of circulating macromolecules, including therapeutic antibodies. Radiation-induced permeability typically peaks within several days, coinciding with vascular damage, including pericyte depletion and disruption of the basal lamina [17]. Interestingly, the infiltration of inflammatory cells, a critical component of the anti-tumor immune response, exhibits similar kinetics, suggesting shared regulatory mechanisms between vascular permeability and immune cell trafficking.

Here, we revisited the use of anti-PD-L1 antibodies as a strategy to potentiate RT’s vaccine-like effects. Consistent with IFNγ-dependent adaptive resistance after radiation, we observed that CTL infiltration was followed by increased PD-L1 expression and subsequent CTL depletion. Importantly, the administration of a single dose of anti-PD-L1 antibody at the peak of PD-L1 expression preserved the CTL infiltrate and enhanced tumor eradication. These findings highlight a critical window of opportunity to disrupt adaptive resistance and harness the full potential of RT-induced anti-tumor immunity. This approach could inform the rational design of combination regimens to maximize the therapeutic benefits of immunoradiotherapy.

## 2. Materials and Methods

### 2.1. Cell Lines and Animal Models

4T1 and CT26 cells (ATCC) were cultured in RPMI-1640 media with 10% FBS (Denville Scientific, Metuchen, NJ, USA), 2 mM L-glutamine, and 100 units/mL penicillin/streptomycin. Negative test results for mycoplasma and a murine virus panel were confirmed for 4T1 and CT26 cell samples by IDEXX RADIL.

Animal studies were approved by the University of Chicago IACUC. Tumors were formed subcutaneously on the right flank of female BALB/c (6–8 weeks old, Envigo, Indianapolis, IN, USA) or NSG mice with 5 × 10^5^ 4T1 or 2.5 × 10^5^ CT26 cells. Tumors were treated after 14 days with 0.2 mg anti-PD-L1 (10F.9G2, BioXCell, Lebanon, NH, USA) in 0.1 mL PBS (pH 7.4) injected through the tail vein or with 10 Gy (IR, 2.5 Gy/min, X-RAD 225Cx, Precision X-Ray) and then anti-PD-L1 after 1, 3, 5, or 7 days. Tumors were measured with calipers along the major (a) and minor (b) axes, and volume was calculated as (a × b2)/2. We randomly assigned tumor mice to the groups and treated them in a blind manner.

### 2.2. In Vitro Inhibitor Treatment

5 × 10^4^ CT26 cells were seeded into 6 well plate and allowed to adhere overnight. The cells were treated with 1 μM of STING inhibitor C-178 (Selleckchem, Houston, TX, USA) or 1 μM of Ruxolitinib (Selleckchem) 2 h before radiation. Cells were irradiated with 10 Gy (60 Co, 7.09 cGy/s, GammaCell, Nordion, Ottawa, ON, Canada), and protein was extracted 3 days after radiation.

### 2.3. Immunodetection

For flow cytometry, tumors excised 2 days after treatment were digested using a Mouse Tumor Dissociation Kit (Miltenyi Biotec., Bergisch Gladbach, Germany), and cells strained through a 70 µm filter into RPMI-1640 medium were pelleted by centrifugation and resuspended in 0.1 mL PBS. After 15 min on ice with FcR block (1.0 μg, BioLegend, San Diego, CA, USA), cells were stained with anti-CD3-Alexa 700 or Pacific Blue (17A2), anti-CD8a-BB515 (53–6.7), anti-CD4-PE/Cy7 (GK1.5), anti-CD45-Pacific Blue or Alexa 700 (30-F11), and anti-CD49b-APC (DX5) fluorescent conjugates (BioLegend) for 45 min at 4 °C. Cells washed with PBS and stained with 10 µg/mL propidium iodide (PI) were analyzed along with costained CompBeads on a Fortessa cytometer (BD) and data analyzed by FlowJo (TreeStar Inc., San Carlos, CA, USA) and Prism (GraphPad, Boston, MA, USA).

For immunohistochemistry, excised tumors were fixed, embedded, sectioned, processed, and stained on a Bond RX with the PDL-1C protocol using anti-PD-L1 (E1L3N, Cell Signaling, Danvers, MA, USA) and Bond Polymer Refine Detection (Leica Biosystems, Deer Park, IL, USA). Tissue sections were imaged at 40× on a Pannoramic SCAN BF (Perkin Elmer, Waltham, MA, USA). PD-L1 staining was quantified with ImageJ (version 1.53h) at six randomly selected regions to obtain mean intensity ± SE.

For immunofluorescence, sections were manually processed and stained with anti-PD-L1 (17952-1-AP, Proteintech, Rosemont, IL, USA, 1:600), anti-CD45 (10-F11, BD, 1:750) or anti-CD8a (4SM15, eBioscience, San Diego, CA, USA, 1:500), and anti-perforin (E3W4I, Cell Signaling, 1:700), each overnight at 4 °C, washed in TBS-Tween 20 (0.05%), stained with anti-rabbit-DyLight594 (Jackson ImmunoResearch, West Grove, PA, USA), anti-rat-647 (Jackson), and DAPI (1 µg/mL), mounted in Prolong Gold (Invitrogen, Carlsbad, CA, USA) and imaged at 40× using an SP5 confocal microscope (Leica Microsystems, Deerfield, IL, USA).

For Western blotting, CT26 cells treated with 10 Gy were analyzed at 1, 3, 5, or 7 days with anti-PD-L1 (ab213480, Abcam, Waltham, MA, USA, 1:1000) and anti-rabbit-HRP (Cytiva, Marlborough, MA, USA, 1:10,000) using anti-actin-HRP as the loading control.

### 2.4. Tracking Antibody Delivery

Anti-PD-L1 (10F.9G2, BioXCell) and anti-CD31 (MEC13.3, Biolegend, San Diego, CA, USA) diluted in PBS pH 8.0 were labeled with NHS-activated DyLight 488, 594, or 633 (Thermo, Waltham, MA, USA) overnight at 4 °C, dialyzed against PBS pH 7.4 at 4 °C for 3 days, and stored at 4 °C.

At 5 days after 4T1 and CT26 tumors were irradiated with 10 Gy, mice were injected i.v. with anti-PD-L1-DyLight 594 (0.2 mg in 0.1 mL PBS) and tumors excised after 30 min. Tumors were embedded in Tissue-Tek O.C.T. Compound (Sakura FineTek, Torrance, CA, USA), frozen at −80 °C, and sectioned at −20 °C. Sections dried at room temperature were rehydrated in PBS, stained with anti-PD-L1-DyLight 488 and anti-CD31-DyLight 633, washed, and imaged with an SP8 confocal microscope (Leica Microsystems).

### 2.5. Quantification and Statistical Analysis

Data were analyzed using Prism 10.0.0 (GraphPad, Boston, CA, USA) and represented as mean ± SE. A comparison of the two groups was carried out using the Mann–Whitney U test. Repeated-measure two-way ANOVA (mixed-model) followed by the Bonferroni post hoc test was used for the analysis of tumor growth curves. *p* ≤ 0.05 was considered statistically significant.

## 3. Results

### 3.1. Anti-PD-L1 Potentiates Radiation Effects

**Figure 1:**
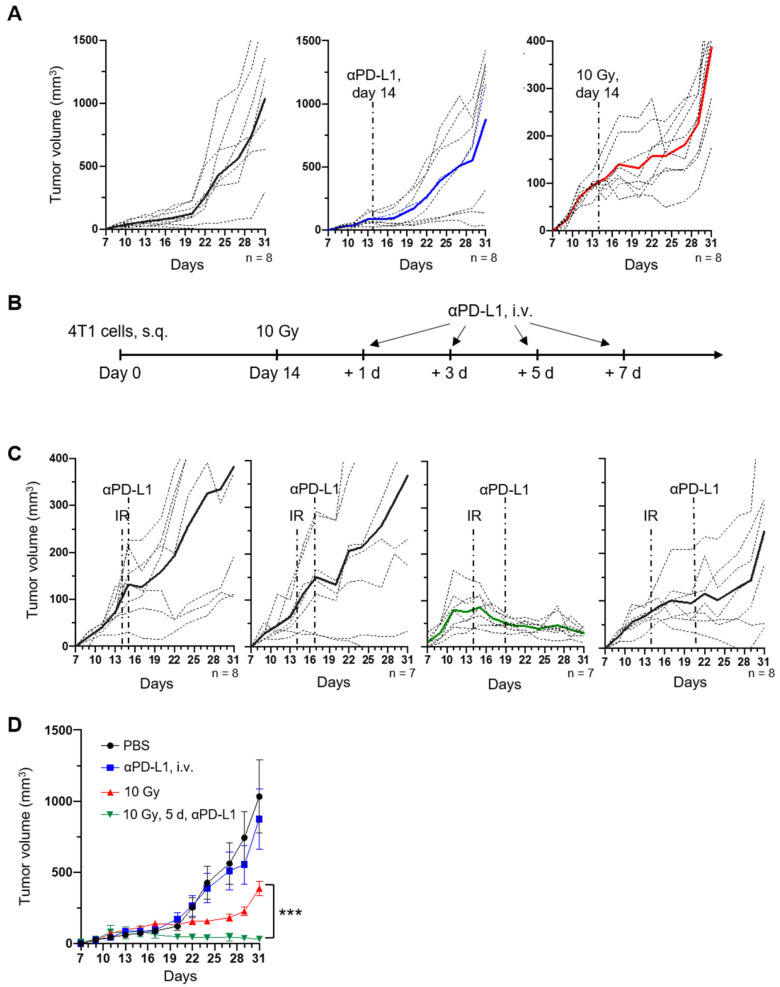
PD-L1 antibody at 5 days after radiation leads to tumor suppression. (**A**) 4T1 tumors were established in BALB/c immunocompetent mice at day 0 and treated at day 14 with PBS, anti-PD-L1, or 10 Gy, showing individual tumor growth profiles (dotted lines) and mean (bold solid lines, black, blue, and red lines corresponding to those shown in (**D**)). (**B**) Schema for combination therapy where tumors irradiated on day 14 are treated with anti-PD-L1 after 1, 3, 5, or 7 days. (**C**) Growth profiles for individual tumors (dotted lines) and mean (bold solid lines, green line corresponding to that shown in (**D**)) for combination treatment, with timing as indicated. (**D**) Mean tumor size for each group comparing controls to optimal combination therapy. *** *p* < 0.01, *n* = 7–8.

In prior studies [18], we observed that circulating 10F.9G2 anti-PD-L1 peaks within minutes after tail vein injection and then returns to nearly baseline by 24 h, offering a means to examine optimal sequencing with ionizing radiation (IR). To evaluate this, on day 0, we injected 4T1 or CT26 carcinoma cells into the right hindlimb of female BALB/c mice, establishing immunocompetent tumor models. Treating tumors on day 14 with a single intravenous injection of 0.2 mg anti-PD-L1 antibody alone did not significantly affect tumor growth kinetics, whereas a single 10 Gy dose of IR induced a transient growth delay (Figure 1A, Supplementary Figure S1A). This highlights the limited efficacy of monotherapy in these models and underscores the need for combinatory strategies to achieve more durable tumor control.

To investigate the impact of sequencing in combination therapy, we treated tumors with 10 Gy IR at 14 days post-injection and subsequently administered anti-PD-L1 at 1, 3, 5, or 7 days after radiation (Figure 1B,C, Supplementary Figure S1B,C). A prolonged tumor growth delay compared to 10 Gy alone was observed only when anti-PD-L1 was administered 5 days post-IR (*p* < 0.05, Figure 1C,D, Supplementary Figure S1C,D). These data suggest that the timing of checkpoint blockade administration is critical for synergizing with RT, potentially reflecting the kinetics of immune cell infiltration and PD-L1 upregulation within the tumor microenvironment.

To confirm the dependence of this effect on functional lymphocytes, we repeated the experiments in immunodeficient NSG mice. Neither 10 Gy IR nor anti-PD-L1 alone or in combination produced a significant growth delay in 4T1 and CT26 tumors in these models (Supplementary Figure S2). These findings reinforce the role of lymphocytes in mediating the observed therapeutic synergy and suggest that adaptive immune mechanisms underlie the delayed tumor growth when combining radiation with checkpoint blockade.

Interestingly, the observed therapeutic window for anti-PD-L1 administration coincides with reported peaks in radiation-induced PD-L1 expression and CTL infiltration. This raises the possibility that delayed anti-PD-L1 administration may protect CTLs from PD-L1-mediated suppression during a critical phase of their effector activity.

### 3.2. PD-L1 Expression and TILs in the Response to Radiation and Combination Therapy

**Figure 2:**
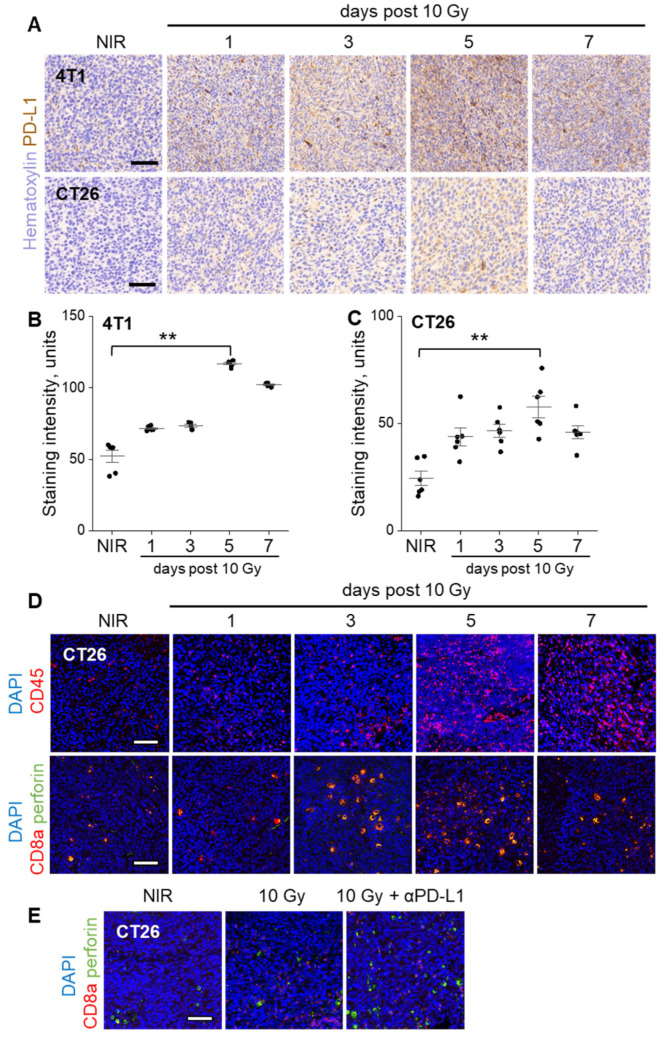
PD-L1 expression and TIL accumulation after radiation. (**A**) PD-L1 immunohistochemistry in 4T1 and CT26 tumors treated with 10 Gy and harvested after 1, 3, 5, or 7 days. Relative quantification of PD-L1 expression in (**B**) 4T1 and (**C**) CT26 tumors. Mean ± SE. (**D**) Tumor sections were stained with anti-CD45 (red) or anti-CD8a (red) and anti-perforin (green) and DAPI (blue). (**E**) Tumors treated with 10 Gy alone or with anti-PD-L1 at 5 days and harvested at 7 days and stained with anti-CD8a (red) and anti-perforin (green). ** *p* < 0.01. Scale bars: 50 µm.

To investigate the dynamics of PD-L1 expression following ionizing radiation (IR), we collected 4T1 and CT26 tumors at 1, 3, 5, or 7 days after a 10 Gy dose and performed anti-PD-L1 immunohistochemistry (Figure 2A–C). IR induced a time-dependent increase in PD-L1 expression in tumors, with both 4T1 and CT26 models showing a significant peak in expression at 5 days post-IR (4T1 ** *p* = 0.022, CT26 ** *p* = 0.022, Figure 2B,C). These findings are consistent with previous reports suggesting that radiation-induced interferon-gamma (IFNγ) released by activated T cells contributes to the upregulation of PD-L1 on tumor and stromal cells. The temporal alignment of PD-L1 expression with other post-radiation immune dynamics highlights the potential for precisely timed therapeutic interventions to target adaptive resistance mechanisms.

To examine the accumulation and activation of cytotoxic T lymphocytes (CTLs) after radiation, CT26 tumors were excised at 1, 3, 5, or 7 days post-IR, fixed, sectioned, and stained with antibodies against CD45 (leukocyte marker), CD8a (CTL marker), and perforin (effector molecule). Tumors displayed heterogeneous immune cell infiltration, with CD45 + cells and CD8a + TILs primarily localized at the periphery of necrotic regions and the tumor capsule. Within the tumor parenchyma, unirradiated tumors exhibited a sparse, unactivated CTL infiltrate, characterized by low levels of perforin expression. By 3 days post-IR, there was a noticeable increase in CD45+ immune cells (Figure 2D), and many CD8a + TILs co-expressed perforin, suggesting early CTL activation.

At 5 days post-IR, the CD45 + immune infiltrate further expanded; however, the density of intact CD8a + TILs began to decline, possibly due to exhaustion or apoptosis triggered by the immunosuppressive tumor microenvironment, including increased PD-L1 expression. By 7 days post-IR, the CD45+ cell density remained elevated, but CD8a + cell density decreased further, indicating a progressive depletion of CTLs.

Importantly, treatment with anti-PD-L1 at day 5 after IR preserved CD8a+ TILs and perforin expression at 7 days post-IR, preventing CTL exhaustion or depletion. Notably, we also observed an increase in CD8a-negative, perforin-positive cells (Figure 2E). These cells may represent natural killer (NK) cells or plasmacytoid dendritic cells (pDCs), which are known to contribute to innate immune responses and cytotoxic activity in certain tumor settings [19,20]. This suggests that anti-PD-L1 therapy not only supports CTL persistence and function but may also enhance the recruitment or activation of other cytotoxic immune cell subsets.

These findings highlight the dynamic interplay between radiation-induced immune responses, adaptive resistance mediated by PD-L1 upregulation, and the potential of anti-PD-L1 therapy to reinvigorate TILs and sustain anti-tumor immunity. Future studies should explore the functional contributions of these non-CTL immune subsets and their role in the therapeutic efficacy of combined RT and checkpoint blockade.

To examine the immune response to combination treatment, 4T1 and CT26 tumors were excised 7 days after 10 Gy, with or without anti-PD-L1 on day 5. Tumors (*n* ≥ 3 per group) were dissociated, and the cell suspension was immunostained for flow cytometric analysis of TILs (Supplementary Figures S3 and S4). Anti-PD-L1 substantially increased CD45 + cells compared to radiation alone (4T1 *p* = 0.11, CT26 * *p* = 0.032) with several TIL subsets appearing increased, including CD45 + CD3 + T cells, the CD4 + and CD8a + T cell subsets and CD49b + natural killer (NK) cells. CT26 tumors contained higher TILs than 4T1 tumors, perhaps reflecting higher immunogenicity, but responses were similar.

### 3.3. Radiation Facilitates Anti-PD-L1 Delivery

**Figure 3:**
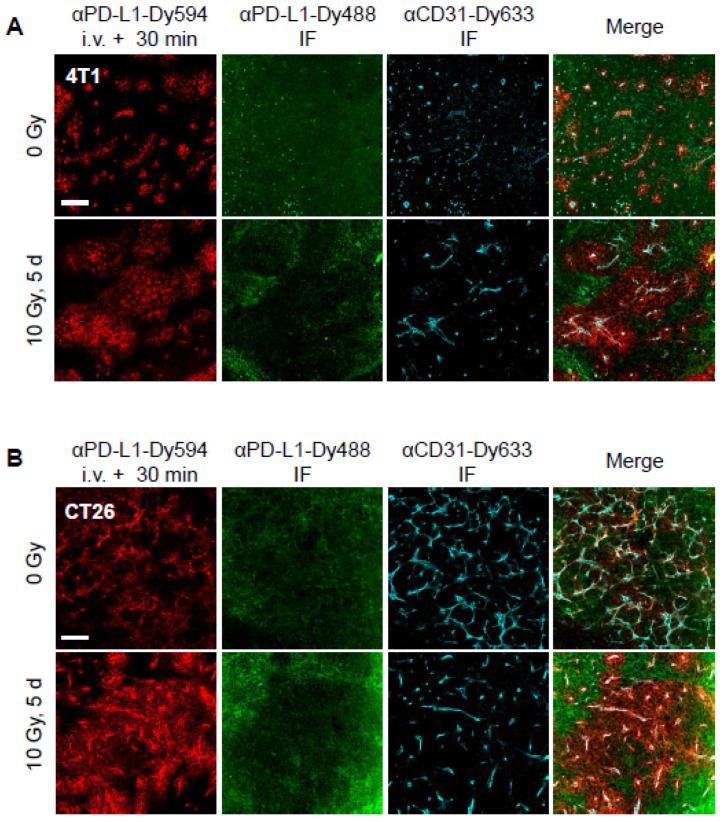
Radiation-induced permeability enhances anti-PD-L1 delivery. Distribution patterns of anti-PD-L1-DyLight 594 (red) in non-irradiated (upper row) or irradiated (lower row) of 4T1 (**A**) and CT26 (**B**) tumors. Staining with anti-PD-L1-DyLight 488 (green) indicates PD-L1 not bound by injected anti-PD-L1 and anti-CD31 indicates vascular endothelium (cyan). Scale bars: 100 µm.

To track antibody delivery and assess its distribution within the tumor microenvironment, mice were injected with anti-PD-L1-DyLight 594, and tumors were excised after 30 min. Tumor sections were subsequently stained with anti-CD31-DyLight 633 to visualize the microvascular endothelium and anti-PD-L1-DyLight 488 to label PD-L1 not reached by anti-PD-L1-DyLight 594 (Figure 3). In both 4T1 (A) and CT26 (B) tumor models, anti-PD-L1-DyLight 594 staining was predominantly restricted to CD31+ perivascular regions in unirradiated tumors, indicating limited extravasation of the antibody from the vasculature. As a result, much of the tumor-associated PD-L1 remained unbound and was readily detectable using anti-PD-L1-DyLight 488.

When anti-PD-L1-DyLight 594 was administered 5 days after a 10 Gy dose of radiation, a marked improvement in antibody extravasation and tissue penetration was observed. In irradiated tumors, anti-PD-L1-DyLight 594 staining extended beyond the CD31+ perivascular regions, showing deeper penetration into the tumor parenchyma. This enhanced delivery masked PD-L1 from detection by anti-PD-L1-DyLight 488, suggesting that a greater proportion of PD-L1 in the tumor was bound by the injected antibody. These findings are consistent with the radiation-induced disruption of the tumor microvascular barrier, which is known to enhance permeability and facilitate the delivery of circulating macromolecules, including therapeutic antibodies.

Notably, the timing of antibody administration post-irradiation appears to be critical for achieving optimal delivery. Prior studies showed that radiation-induced vascular permeability peaks within several days after treatment, coinciding with damage to endothelial cells, pericytes, and the basal lamina. The increased extravasation observed at day 5 post-IR suggests that this window represents a period of maximal vascular permeability, enabling deeper penetration of therapeutic antibodies into the tumor microenvironment.

This enhanced delivery may also improve the therapeutic efficacy of anti-PD-L1 by enabling a more comprehensive blockade of tumor-associated PD-L1, particularly in regions distal to the vasculature where immune effector cells may encounter unbound PD-L1. Future experiments could explore whether repeated or higher doses of antibody during this permeability window further enhance delivery and therapeutic outcomes. Additionally, studies to investigate the spatial relationship between antibody distribution and immune cell infiltration could provide insights into the mechanisms by which improved delivery translates to increased anti-tumor immunity.

### 3.4. TILs Determine PD-L1 Expression After Radiation

**Figure 4:**
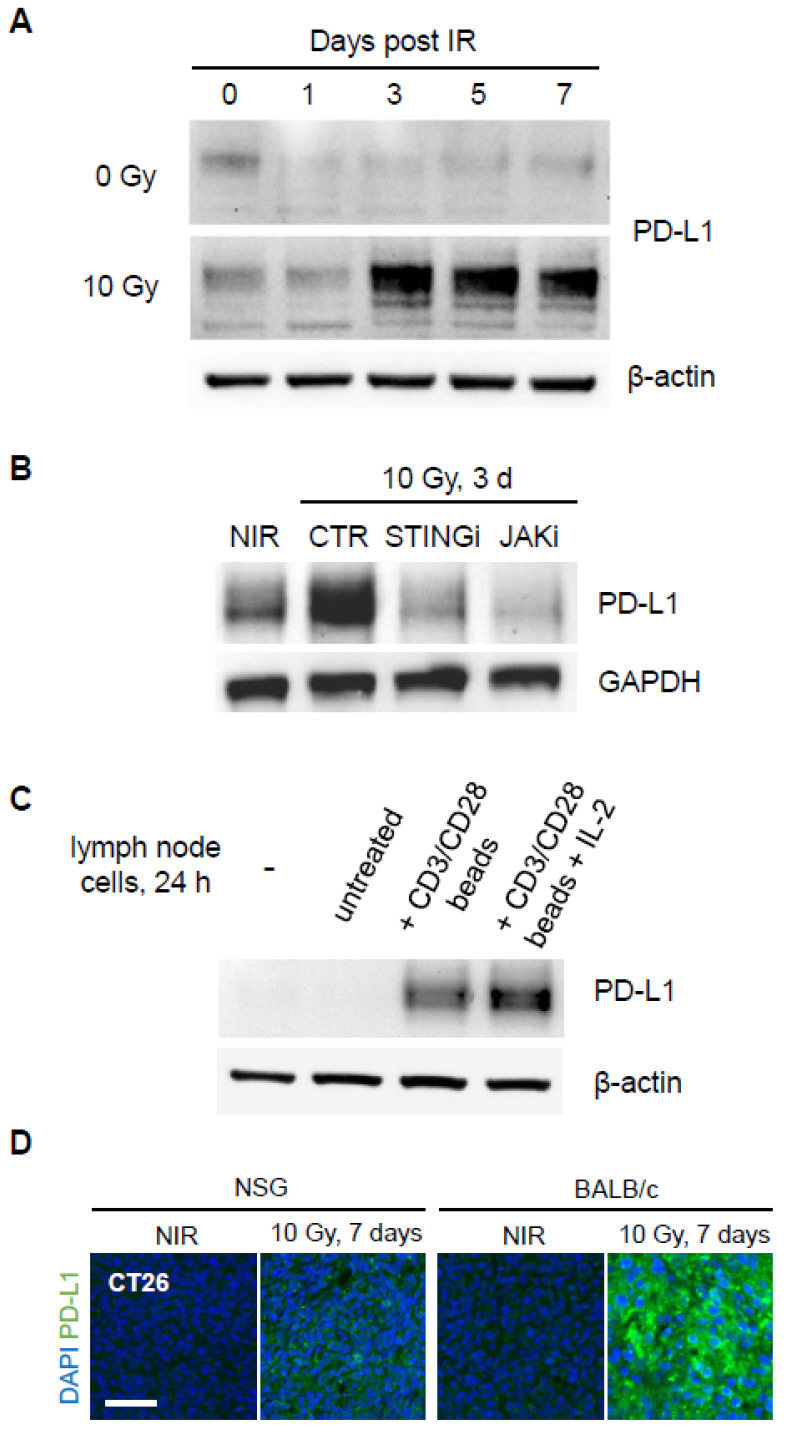
Intrinsic vs. paracrine PD-L1 upregulation in response to radiation. (**A**) CT26 cells were treated with 0 or 10 Gy and incubated for the indicated time, and then cell lysates were examined by anti-PD-L1 immunoblot. (**B**) CT26 cells were treated with 1 μΜ STING or JAK inhibitor prior to 0 or 10 Gy irradiation and cell lysates isolated after 3 days for anti-PD-L1 immunoblot. (**C**) CT26 cells were treated with the indicated agents or coincubated for 24 h with mouse lymph node cells, untreated, activated with anti-CD3/CD28 beads, or activated with anti-CD3/CD28 beads with IL-2 and lysates isolated for anti-PD-L1 immunoblot. (**D**) CT26 tumors in BALB/c or NSG mice were treated with a single 10 Gy dose, isolated after 7 days, stained with anti-PD-L1 antibody, and imaged by immunofluorescence. Scale bar: 100 µm.

To evaluate cell-intrinsic effects on PD-L1 expression, CT26 cells were treated with 0 or 10 Gy, incubated for up to 7 days, and lysates analyzed by Western blot (Figure 4A). PD-L1 was not induced until day 3, suggesting an indirect and/or delayed effect of radiation. Treatment with 10 Gy in the presence of STING or JAK inhibitors blocked PD-L1 induction at 3 days (Figure 4B), suggesting a role for autocrine signaling. The co-culture of CT26 cells with mouse lymph node cells activated with anti-CD3/CD28 beads as a source of cytokines rapidly induced PD-L1 (Figure 4C). To extend this comparison in vivo, CT26 tumors formed for 14 days in BALB/c or immunodeficient NSG mice were treated with 10 Gy and tumors excised after 5 days for analysis of PD-L1 expression (Figure 4D). Although PD-L1 was induced in NSG mice, the levels were significantly lower compared to those in BALB/c mice. This suggests that both cell-intrinsic signaling and the effects of infiltrating activated TILs, contributing to PD-L1 upregulation in vivo.

## 4. Discussion

The anti-tumor immune response induced by radiation is typically short-lived, as tolerance is rapidly restored via rebound immunosuppression, a phenomenon well-documented in both preclinical and clinical studies [21]. Among the factors that induce PD-L1 in the tumor microenvironment after radiation, the most potent may be IFNγ released by activated TILs themselves [11,12]. Overcoming this adaptive resistance is crucial, as it may unleash a sustained anti-tumor immune response following radiation therapy, potentially leading to durable tumor elimination [22]. Clinical trials showed that the success of combining checkpoint blockade immunotherapy (CBI) with radiation therapy hinges on our understanding the temporal dynamics of adaptive resistance. Our findings align with this principle, emphasizing the importance of timing PD-L1 blockade to maximize therapeutic efficacy.

We identified a relatively short interval after irradiation, during which targeting PD-1/PD-L1 signaling was most effective in disrupting adaptive resistance. This window coincided with the peak of PD-L1 expression, driven by the inflammatory infiltrate. Clinical studies support this concept. For instance, the PACIFIC trial demonstrated that durvalumab, an anti-PD-L1 antibody, significantly prolonged progression-free survival when administered shortly after chemoradiotherapy in patients with stage III non-small-cell lung cancer (NSCLC) [23]. This suggests that the inflammatory and immune-permissive environment induced by radiation may prime tumors for enhanced responsiveness to CBIs, particularly when treatment is initiated within a critical window. However, the optimal timing of CBI initiation remains under investigation in ongoing trials, including NCT03391869, which explores the timing of CBIs after radiation in metastatic NSCLC.

Our findings add to a growing body of evidence suggesting that timing is critical in determining the success of radiation–CBI combinations. For example, in preclinical models of colon and breast cancer, it has been observed that CBIs administered either too early or too late relative to radiation fail to achieve maximal efficacy, potentially due to inadequate TIL activation or the resolution of radiation-induced inflammatory signals [24]. This underscores the need for carefully defined protocols that consider both the dynamics of immune activation and PD-L1 expression following radiation.

Mechanistically, our results indicate that PD-L1 induction is closely linked to TIL accumulation. This effect was blunted in NSG mice lacking T, B, and NK cells, underscoring the importance of TILs in shaping the immune response. By disrupting the negative feedback loop through anti-PD-L1 antibody treatment, we observed increased TIL infiltration and subsequent tumor elimination. These findings are consistent with recent reports that highlight the role of adaptive immunity in regulating tumor resistance to therapy [25]. Translating these findings into clinical practice could involve identifying circulating or tissue markers of adaptive resistance to guide the timing of checkpoint blockade therapy after irradiation, an approach supported by emerging clinical data [26,27].

Another critical factor influencing the timing of CBI efficacy is radiation-induced vascular permeability. Similar to PD-L1 accumulation, permeability changes occur over several days after radiation. The transient disruption of pericyte coverage and extracellular matrix not only enhances macromolecular delivery but also facilitates leukocyte extravasation, amplifying the effects of well-timed CBI [17]. Clinical evidence supports this, with studies suggesting that radiation-induced vascular remodeling may enhance drug delivery and immune cell infiltration, particularly when combined with CBI. Trials such as NCT02303990, which examined pembrolizumab with radiation in metastatic NSCLC or melanoma, highlighted the potential for this synergy to improve outcomes. Moreover, novel approaches such as combining radiation with vascular-targeting agents may further augment immune infiltration and therapy efficacy, as demonstrated in recent preclinical studies [28,29].

In summary, we propose a strategy for sequencing single doses of ionizing radiation with immune checkpoint blockade therapy to disrupt adaptive resistance and achieve robust anti-tumor responses in multiple tumor models. These findings complement existing clinical trials that highlight the importance of timing CBIs relative to radiation to maximize therapeutic benefit. Future clinical applications of similar treatment regimens, guided by biomarkers of adaptive resistance, vascular remodeling, and immune activation, may provide greater benefits to a broader patient population. Furthermore, understanding the interplay between radiation and CBIs may inform personalized treatment schedules that optimize efficacy while minimizing toxicity.

## 5. Conclusions

Our findings underscore the importance of strategically timing radiation to local tumors to enhance the targeted delivery of checkpoint blockade immunotherapy by leveraging increased vascular permeability and PD-L1 expression within the tumors. This approach offers a promising strategy to amplify the effects of external beam radiotherapy by protecting activated tumor-infiltrating lymphocytes (TILs) and strengthening the anti-tumor immune response. This study provides critical insights for optimizing the timing and treatment schedule of combined immune checkpoint blockade (ICB) and radiation therapy to achieve superior clinical outcomes in cancer treatment.

## Data Availability

All data relevant to the study are included in the article or uploaded as Supplementary Materials.

## Supplementary Materials

The following supporting information can be downloaded at: https://www.mdpi.com/article/10.3390/cancers17030391/s1, Figure S1. PD-L1 antibody at 5 days after radiation leads to tumor suppression. (A) CT26 tumors were established in BALB/c immunocompetent mice at day 0 and treated at day 14 with PBS, anti-PD-L1, or 10 Gy, showing individual tumor growth profiles (dotted lines) and mean (bold solid lines, black, blue, and red lines corresponding to those shown in (D)). (B) Schema for combination therapy where tumors irradiated at day 14 are treated with anti-PD-L1 after 1, 3, 5, or 7 days. (C) Growth profiles for individual tumors (dotted lines) and mean (bold solid lines, green line corresponding to that shown in (D)) for combination treatment, with timing as indicated. (D) Mean tumor size for each group comparing controls to optimal combination therapy. *** *p* < 0.01, *n* = 6–8. Figure S2. Radiation and anti-PD-L1 are ineffective in immunodeficient NSG mice. Individual (dotted lines) and mean (bold solid lines, black, blue, red, and green lines corresponding to those shown in (C) and (D)) growth profiles for 4T1 (A) and CT26 (B) tumors treated with PBS, anti-PD-L1, 10 Gy, or combination treatment with anti-PD-L1 injected 5 days after 10 Gy. Mean 4T1 (C) and CT26 (D) tumor growth profiles of the four treatment groups. ns *p* > 0.05, *n* = 7–8. Figure S3. TILs are recruited to tumors by PD-L1 antibody treatment at 5 days post-IR. Flow cytometry was used to quantify the percentage of TILs and key TIL subsets in 4T1 and CT26 tumors treated with 10 Gy alone or with anti-PD-L1 after 5 days. Percentage of total viable cells in samples representing CD45+ infiltrating cells, CD45+ CD3+ T cells, CD45+ CD3+ CD4+ helper T cells, CD45+ CD3+ CD8+ cytotoxic T cells, and CD45+ CD49b+ natural killer cells. Mean percentage ± SEM, n = 3–4 tumors per group, * *p* < 0.05, ns *p* > 0.05. Figure S4. Representative flow cytometry data and gating used in Supplementary Figure S3 for 4T1 tumor treated with 10 Gy (A) or 10 Gy and anti-PD-L1 (B) and CT26 tumor treated with 10 Gy (C) or 10 Gy and anti-PD-L1 (D). Figure S5. Whole gel images and band intensities of Figure 4A,B.
