# Supplementary material for: Timing Anti-PD-L1 Checkpoint Blockade Immunotherapy to Enhance Tumor Irradiation
Source: Cancers (Basel). 2025 Jan 24;17(3):391. doi: 10.3390/cancers17030391 (PMC11815760; doi:10.3390/cancers17030391)
Supplement: Supplementary file 1 [file cancers-17-00391-s001.zip › cancers-3410546-supplementary.pdf]

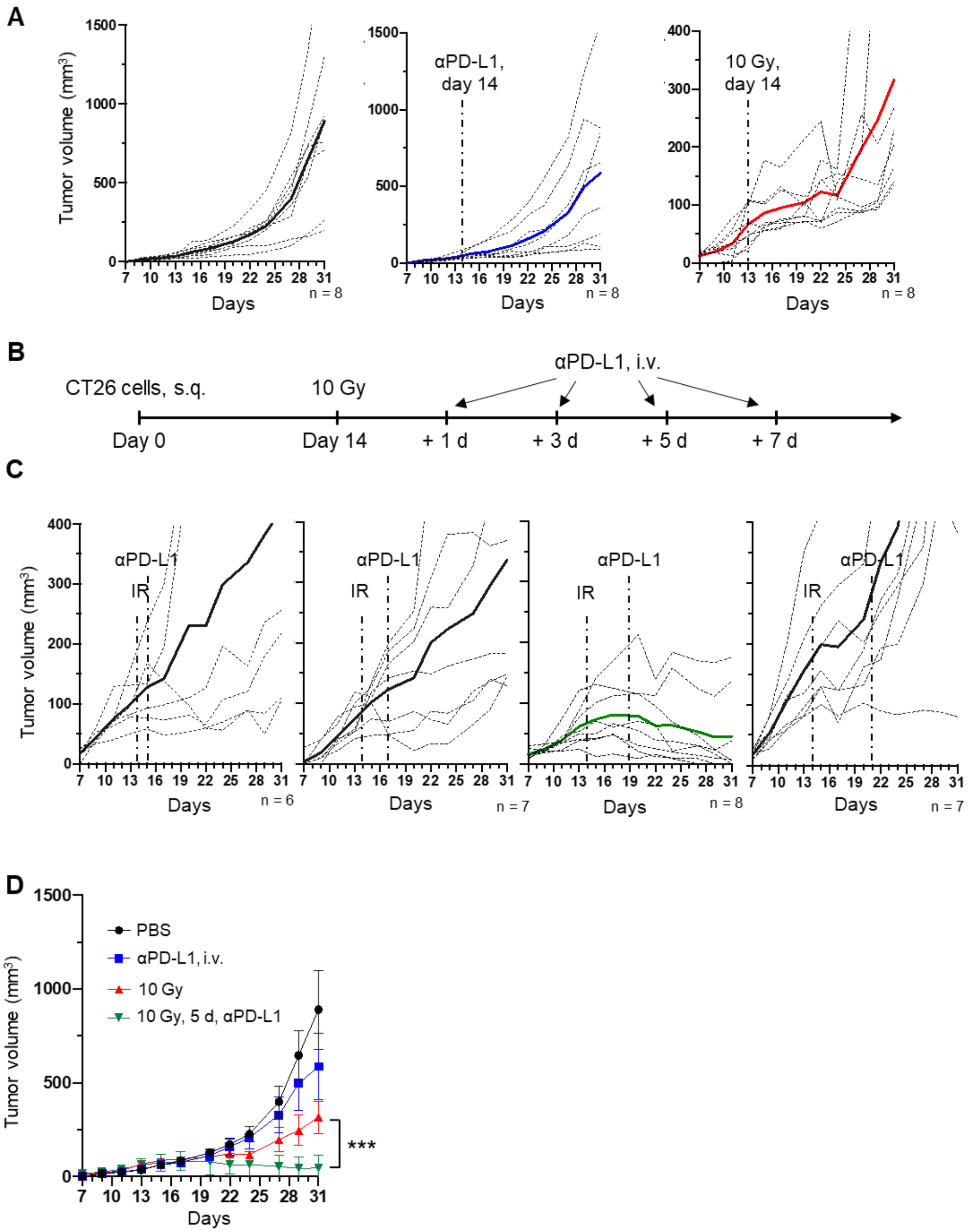


**Supplemental Figure S1.** PD-L1 antibody at 5 days after radiation leads to tumor suppression. (**A**) CT26 tumors were established in BALB/c immunocompetent mice at day 0 and treated at day 14 with PBS, anti-PD-L1, or 10 Gy, showing individual tumor growth profiles (dotted lines) and mean (bold solid lines, black, blue, and red lines corresponding to those shown in (**D**)). (**B**) Schema for combination therapy where tumors irradiated at day 14 are treated with anti-PD-L1 after 1, 3, 5, or 7 days. (**C**) Growth profiles for individual tumors (dotted lines) and mean (bold solid lines, green line corresponding to that shown in (**D**)) for combination treatment, with timing as indicated. (D) Mean tumor size for each group comparing controls to optimal combination therapy. *** $p < 0.01$, $n = 6$-8.

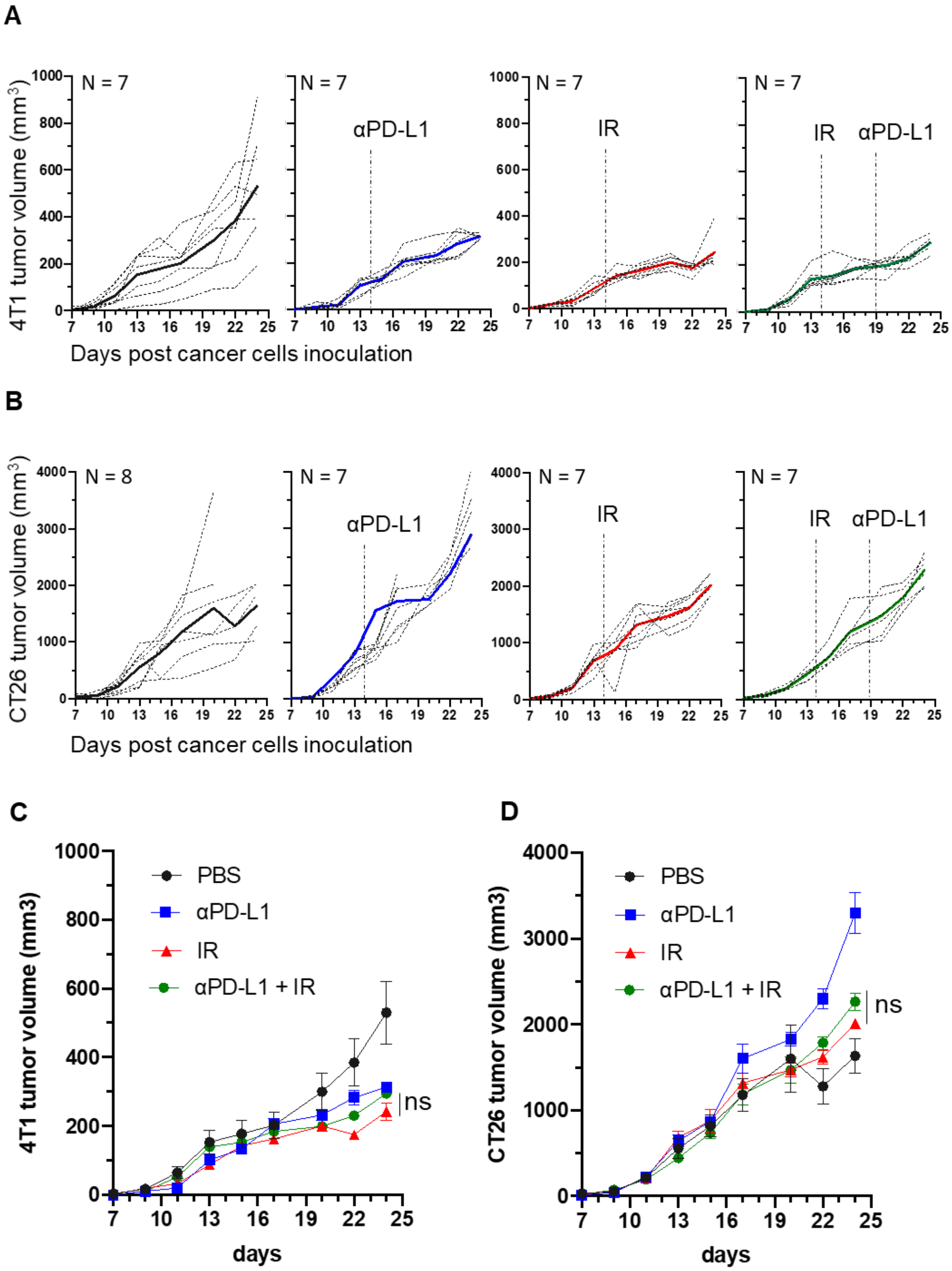


**Supplemental Figure S2.** Radiation and anti-PD-L1 are ineffective in immunodeficient NSG mice. Individual (dotted lines) and mean (bold solid lines, black, blue, red, and green lines corresponding to those shown in (**C**) and (**D**)) growth profiles for 4T1 (**A**) and CT26 (**B**) tumors treated with PBS, anti-PD-L1, 10 Gy, or combination treatment with anti-PD-L1 injected 5 days after 10 Gy. Mean 4T1 (**C**) and CT26 (**D**) tumor growth profiles of the four treatment groups. ns $p > 0.05$, $n = 7–8$.

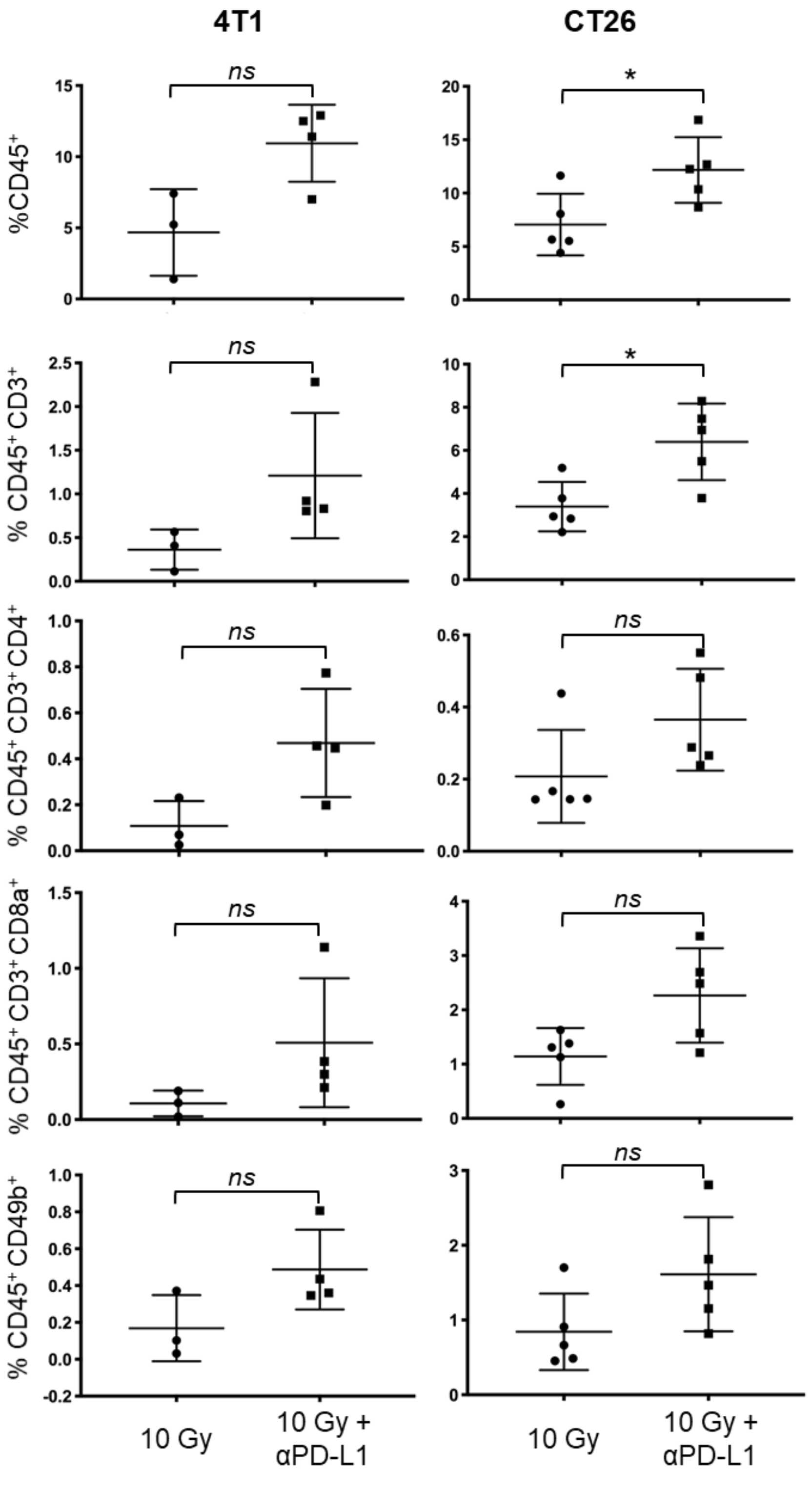


**Supplemental Figure S3.** TILs are recruited to tumors by PD-L1 antibody treatment at 5 days post-IR. Flow cytometry was used to quantify the percentage of TILs and key TIL subsets in 4T1 and CT26 tumors treated with 10 Gy alone or with anti-PD-L1 after 5 days. Percentage of total viable cells in samples representing CD45+ infiltrating cells, CD45+ CD3+ T cells, CD45+ CD3+ CD4+ helper T cells, CD45+ CD3+ CD8+ cytotoxic T cells, and CD45+ CD49b+ natural killer cells. Mean percentage ± SEM, n = 3–4 tumors per group, * $p < 0.05$, ns $p > 0.05$.

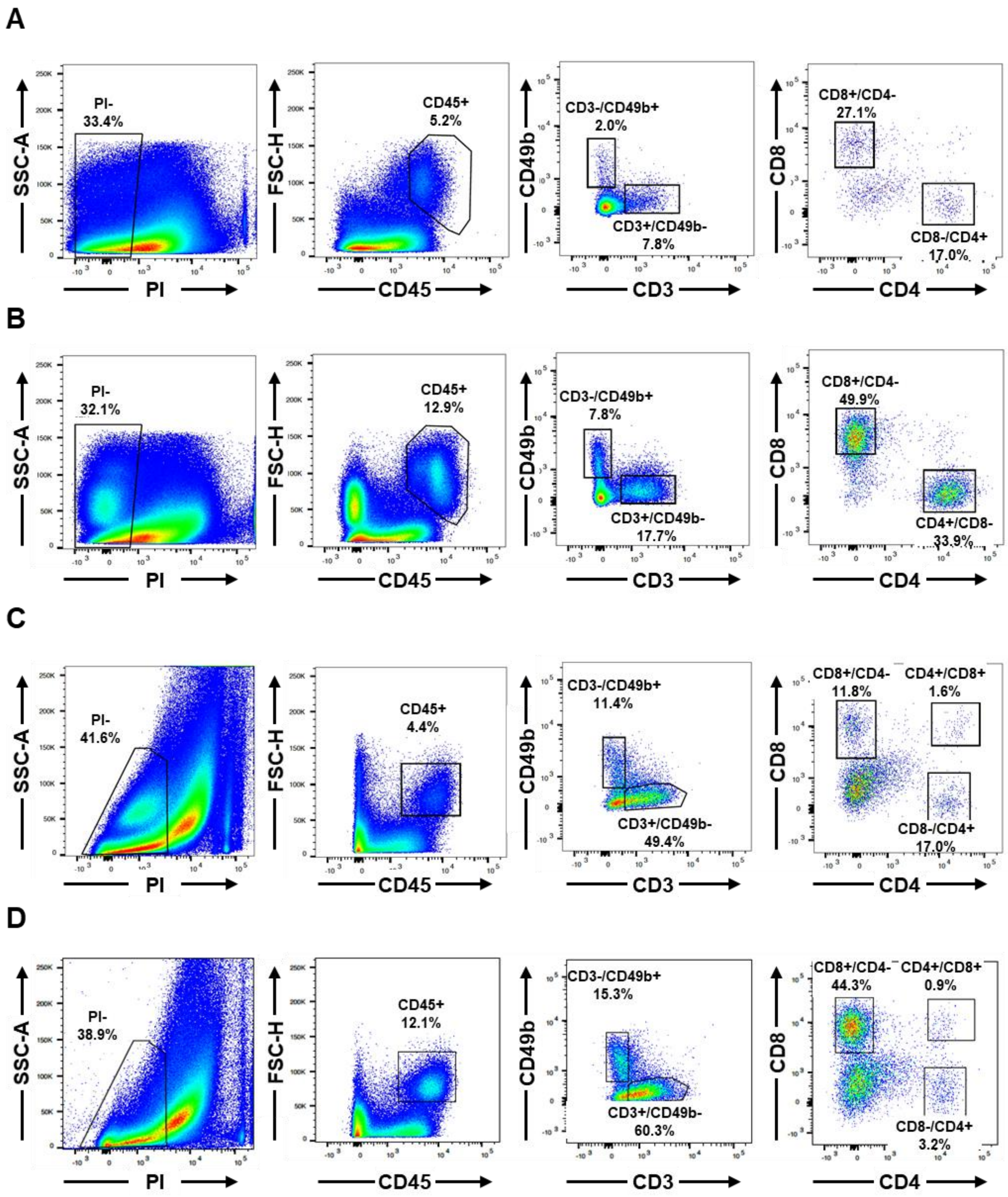


**Supplemental Figure S4.** Representative flow cytometry data and gating used in Supplementary Figure S3 for 4T1 tumor treated with 10 Gy (**A**) or 10 Gy and anti-PD-L1 (**B**) and CT26 tumor treated with 10 Gy (**C**) or 10 Gy and anti-PD-L1 (**D**).

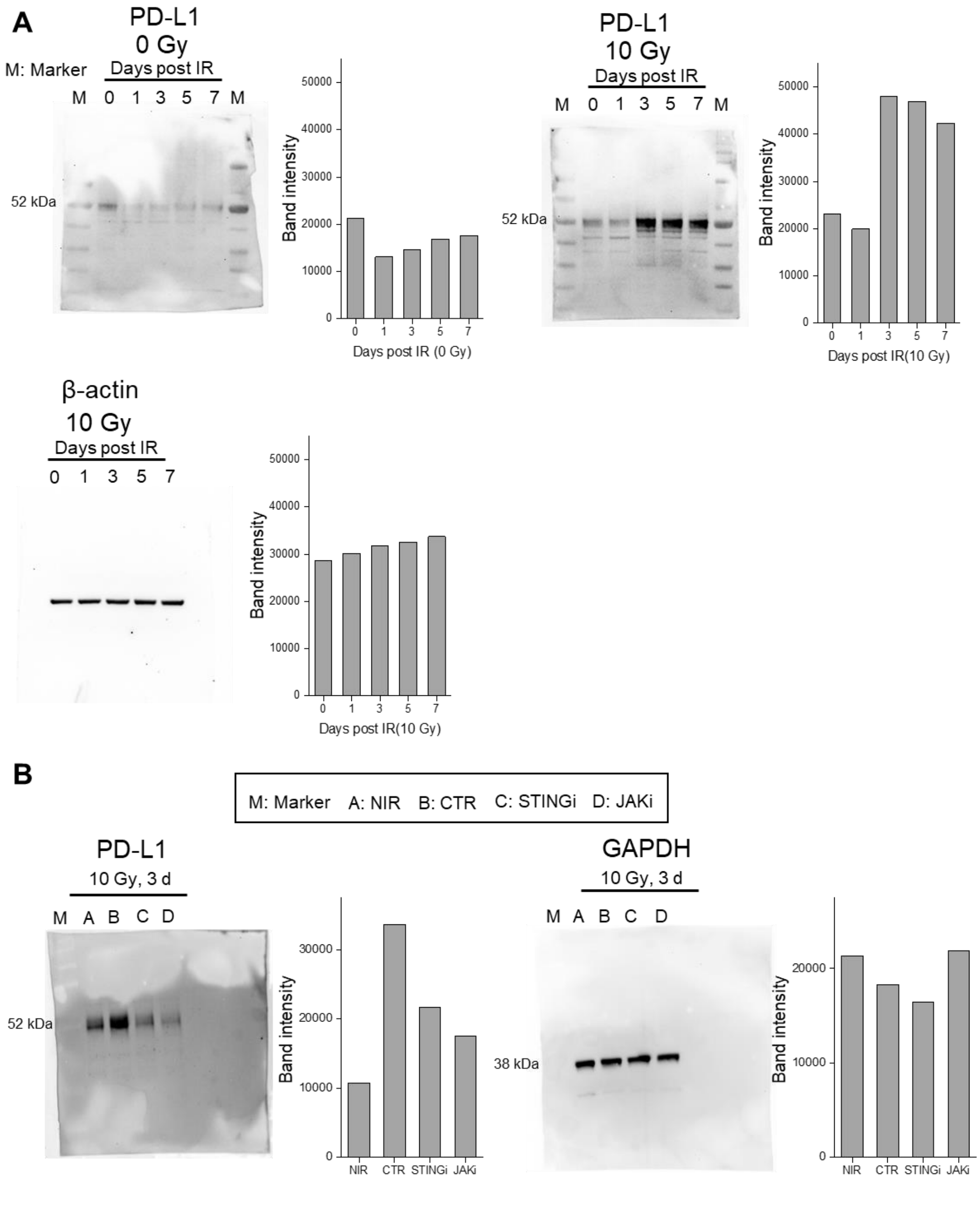


**Supplemental Figure S5.** Whole gel images and band intensities of figure 4A (**A**) and B (**B**).
